# Supplementary material for: Evolutionary Stability of Small Molecular Regulatory Networks That Exhibit Near-Perfect Adaptation
Source: Biology (Basel). 2023 Jun 9;12(6):841. doi: 10.3390/biology12060841 (PMC10295370; doi:10.3390/biology12060841)
Supplement: Supplementary file 1 [file biology-12-00841-s001.zip › Supplementary Information Revised Version.pdf]

**Supplementary Information**

*Evolutionary Stability of Small Molecular Regulatory Networks that Exhibit Near-Perfect Adaptation*

Rajat Singhania and John J. Tyson

**Outline:**

**Supplementary Text S1.** *A Catalogue of Mechanisms for Robust Perfect Adaptation and Near Perfect Adaptation*

**Supplementary Table S1.** The *pure* signaling motifs.

**Supplementary Table S2.** NFLB-1 topologies (1X3X3X) macro-mutate predominantly into high-scoring IFFL-1 + NFLB-1 topologies (1X3X31).

**Supplementary Table S3.** NFLB-3 topologies (XXX331) macro-mutate predominantly into high-scoring IFFL-1 + NFLB-1 topologies (1X3X31).

**Supplementary Table S4.** NFLB-2 topologies (3X1X3X) macro-mutate predominantly to high-scoring IFFL-1 + NFLB-1 topologies (1X3X31).

**Supplementary Table S5.** NFLB-4 topologies (XXX133) macro-mutate predominantly to high-scoring IFFL-1 + NFLB-1 topologies (1X3X31).

**Supplementary Table S6.** The percentage change in average score <*Z*> going from an *uncoupled* NFLB-1 topology to a coupled NFLB-1 + IFFL-1 topology.

**Supplementary Table S7.** The percentage change in average score <*Z*> going from an *uncoupled* NFLB-2 topology to a coupled NFLB-2 + IFFL-4 topology.

**Supplementary Table S8.** The percentage change in average score <*Z*> going from an *uncoupled* NFLB-3 topology to a coupled NFLB-3 + IFFL-1 topology.

**Supplementary Table S9.** The percentage change in average score <*Z*> going from an *uncoupled* NFLB-4 topology to a coupled NFLB-4 + IFFL-4 topology.

**Supplementary Table S10.** The percentage changes in average score < *Z* > going from an *uncoupled* IFFL-1 topology to a coupled IFFL-1 + NFLB-1 topology.

**Supplementary Table S11.** The percentage changes in average score < *Z* > going from an *uncoupled* IFFL-1 topology to a coupled IFFL-1 + NFLB-3 topology.

**Supplementary Table S12.** The percentage changes in average score < *Z* > going from an *uncoupled* IFFL-1 topology to a coupled IFFL-1 + NFLB-1 + NFLB-3 topology.

**Supplementary Table S13.** The percentage changes in average score < *Z* > adding an NFLB-3 topology to a coupled IFFL-1 + NFLB-1 topology.

**Supplementary Table S14.** The percentage changes in average score < *Z* > adding an NFLB-1 topology to a coupled IFFL-1 + NFLB-3 topology.

**Supplementary Table S15.** The percentage changes in average score < *Z* > going from an *uncoupled* IFFL-4 topology to a coupled IFFL-4 + NFLB-2 topology.

**Supplementary Table S16.** The percentage changes in average score < *Z* > going from an *uncoupled* IFFL-4 topology to a coupled IFFL-4 + NFLB-4 topology.

**Supplementary Table S17.** The percentage changes in average score < *Z* > going from an *uncoupled* IFFL-4 topology to a coupled IFFL-4 + NFLB-2 + NFLB-4 topology.

**Supplementary Table S18.** The percentage changes in average score < *Z* > adding an NFLB-4 topology to a coupled IFFL-4 + NFLB-2 topology.

**Supplementary Table S19.** The percentage changes in average score < *Z* > adding an NFLB-2 topology to a coupled IFFL-4 + NFLB-4 topology.

**Supplementary Table S20.** Mean values of the six interaction coefficients from all high-scoring samples of IFFL-1 topologies.

**Supplementary Table S21.** Mean values of the six interaction coefficients from all high-scoring samples of IFFL-4 topologies.

**Supplementary Text S1.** *A Catalogue of Mechanisms for Robust Perfect Adaptation and Near Perfect Adaptation*

Each motif is displayed as a reaction network among four species: S, P, Q and R; where S is the ‘signal’ and R is the ‘response’. (In some cases, P and R are combined into one species.) For robust perfect adaptation (RPA), the steady-state concentration of R (i.e., $R_{ss}$) is independent of the signal strength $S$, regardless of the kinetic parameter values (provided there exists a steady state solution with all positive concentrations). For near-perfect adaptation (NPA), $R_{ss}$ depends only weakly of $S$. For many proposed mechanisms, RPA depends on an assumption that particular chemical reactions proceed at a constant rate, independent of the concentration of the reactant. These reactions are indicated by the symbol (o⟶), and the constant rate of the reaction is denoted by $V$. For the ‘standard’ reactions in the motifs (symbolized by ⟶), the reaction rates are given by the law of mass action, with positive rate constants $k_i$. The role of the ‘signal’ is often expressed by an arbitrary, monotone increasing function $F(S)$; e.g., 1+S, or $\frac{S}{1+S}$, or $\frac{S^n}{1+S^n}$. In cases where Q inhibits a reaction, the rate of the reaction is multiplied by $\frac{1}{1+Q}$, but other monotone decreasing functions would work equally well. In motif #6 below, the T-shaped reaction arrow indicates reversible binding of P + R to yield the complex Q, with forward rate constant $k_{3f}$ and reverse rate constant $k_{3r}$. The Y-shaped connector in motif #11 denotes irreversible binding.

| # | Mechanism | Differential Equations | Steady-State | References |
|---|---|---|---|---|
| 1 | 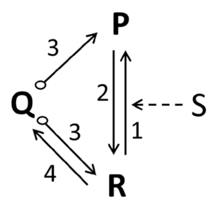 | $\frac{dP}{dt} = k_1SR - k_2P + (1-\alpha)V_3$ <br> $\frac{dQ}{dt} = k_4R - V_3$ <br> $\frac{dR}{dt} = \alpha V_3 + k_2P - k_1SR - k_4R$ | $P = \frac{V_3}{k_2}\left(1 - \alpha + \frac{k_1S}{k_4}\right)$ <br> $R = \frac{V_3}{k_4}$ <br> $P + Q + R = \text{const} > 0$ | Barkai & Leibler (1997) <br> Khammash (2021) |
| 2 | 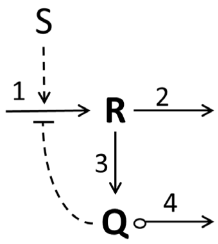 | $\frac{dQ}{dt} = k_3R - V_4$ <br> $\frac{dR}{dt} = \frac{k_1F(S)}{1+Q} - k_2R - k_3R$ | $1 + Q = \frac{k_3k_1F(S)}{V_4(k_2+k_3)} > 1$ <br> $R = \frac{V_4}{k_3}$ | Yi et al. (2000) <br> “integral feedback” |

| | | | |
|---|---|---|---|
| 3 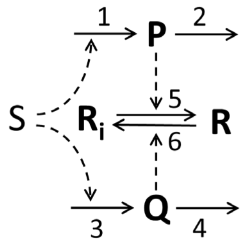  | $\frac{dP}{dt} = k_1F(S) - k_2P$<br>$\frac{dQ}{dt} = k_3F(S) - k_4Q$<br>$\frac{dR}{dt} = k_5P(R_\mathrm{T} - R) - k_6QR$ | $P = \frac{k_1F(S)}{k_2}$<br>$Q = \frac{k_3F(S)}{k_4}$<br>$R = \frac{R_\mathrm{T}}{1 + \frac{k_2k_3k_6}{k_1k_4k_5}}$ | Levchenko & Iglesias (2002) |
| 4 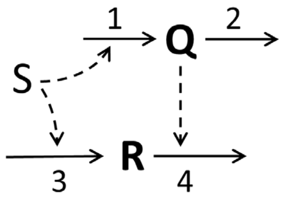  | $\frac{dQ}{dt} = k_1F(S) - k_2Q$<br>$\frac{dR}{dt} = k_3F(S) - k_4QR$ | $Q = \frac{k_1F(S)}{k_2}$<br>$R = \frac{k_2k_3}{k_1k_4}$ | Tyson et al. (2003) (Fig. 1d)<br><br>"Sniffer" |
| 5 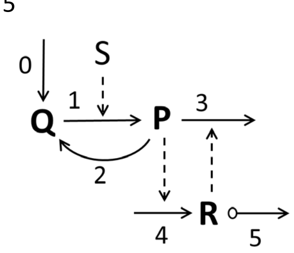  | $\frac{dP}{dt} = k_1SQ - k_2P - k_3RP$<br>$\frac{dQ}{dt} = k_0 - k_1SQ + k_2P$<br>$\frac{dR}{dt} = k_4P - V_5$ | $P = \frac{V_5}{k_4}$<br>$Q = \frac{k_0k_4 + k_2V_5}{k_4k_1S}$<br>$R = \frac{k_0k_4}{k_3V_5}$ | Behar et al. (2007) (Model IVA) |
| 6 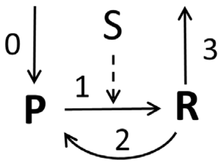  | $\frac{dP}{dt} = k_0 - k_1F(S)P + k_2R$<br>$\frac{dR}{dt} = k_1F(S)P - k_2R - k_3R$ | $P = \frac{k_0(k_2 + k_3)}{k_3k_1F(S)}$<br>$R = \frac{k_0}{k_3}$ | Hao et al. (2007)<br><br>Drengstig et al. (2008) |
| 7 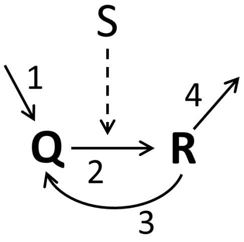  | $\frac{dQ}{dt} = k_1 - k_2F(S)Q + k_3R$<br>$\frac{dR}{dt} = k_2F(S)Q - k_3R - k_4R$ | $Q = \frac{k_1}{k_2F(S)}\left(1 + \frac{k_3}{k_4}\right)$<br>$R = \frac{k_1}{k_4}$ | Francois & Siggia (2008) (Fig. 4) |

| | | | | |
|---|---|---|---|---|
| 8 | 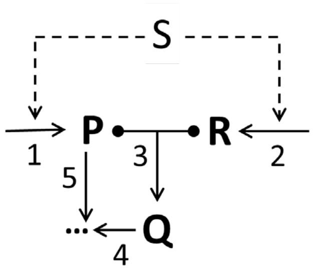 | $\frac{dP}{dt} = k_1F(S)$ $-k_{3\mathrm{f}}PR + k_{3\mathrm{r}}Q - k_5P$<br>$\frac{dQ}{dt} = k_{3\mathrm{f}}PR - k_{3\mathrm{r}}Q - k_4Q$<br>$\frac{dR}{dt} = k_2F(S) - k_{3\mathrm{f}}PR + k_{3\mathrm{r}}Q$ | $P = \frac{(k_1 - k_2)F(S)}{k_5}$<br>$Q = \frac{k_2F(S)}{k_4}$<br>$R = \frac{k_2k_5(k_{3\mathrm{r}} + k_4)}{k_4(k_1 - k_2)k_{3\mathrm{f}}}$ | Francois & Siggia (2008) (Fig. 7) |
| 9 | 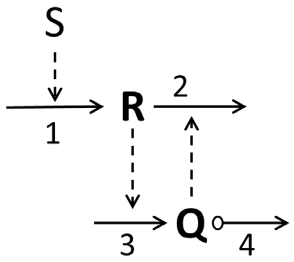 | $\frac{dQ}{dt} = k_3R - V_4$<br>$\frac{dR}{dt} = k_1F(S) - k_2QR$ | $Q = \frac{k_1F(S)}{k_2} \cdot \frac{k_3}{V_4}$<br>$R = \frac{V_4}{k_3}$ | Behar et al. (2007) (Model I)<br><br>Ni et al. (2009) (Fig. 6a) |
| 10 | 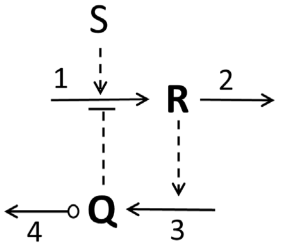 | $\frac{dQ}{dt} = k_3R - V_4$<br>$\frac{dR}{dt} = \frac{k_1F(S)}{1 + Q} - k_2R$ | $1 + Q = \frac{k_1F(S)}{k_2} \cdot \frac{k_3}{V_4} > 1$<br>$R = \frac{V_4}{k_3}$ | Ni et al. (2009) (Fig. 6b) |
| 11 | 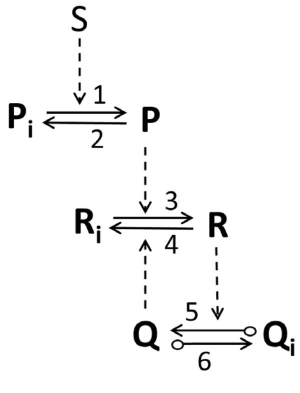 | $\frac{dP}{dt} = k_1S(1 - P) - k_2P$<br>$\frac{dQ}{dt} = k_5R - k_6$<br>$\frac{dR}{dt} = k_3P(1 - R) - k_4QR$ | $P = \frac{k_1S}{k_1S + k_2}$<br>$Q = \frac{k_3(k_5 - k_6)}{k_4k_6}\left(\frac{k_1S}{k_1S + k_2}\right)$<br>$R = \frac{k_6}{k_5} < 1$ | Ma et al. (2009) (Fig. 3a)<br><br>"negative feedback loop with buffer" |

| | | | |
|---|---|---|---|
| 12<br>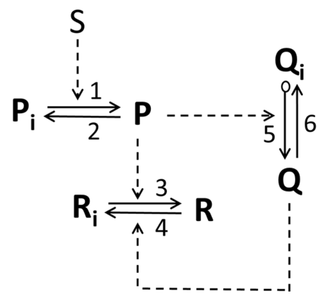 | $\frac{dP}{dt} = k_1S(1-P) - k_2P$<br>$\frac{dQ}{dt} = k_5P - k_6Q$<br>$\frac{dR}{dt} = k_3P(1-R) - k_4QR$ | $P = \frac{k_1S}{k_1S + k_2}$<br>$Q = \frac{k_5}{k_6}\left(\frac{k_1S}{k_1S + k_2}\right)$<br>$R = \frac{1}{1 + \frac{k_4k_5}{k_3k_6}}$ | Ma et al. (2009) (Fig. 3b)<br>“incoherent feedforward loop” |
| 13<br>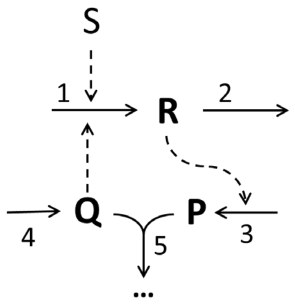 | $\frac{dP}{dt} = k_3R - k_5PQ$<br>$\frac{dQ}{dt} = k_4 - k_5PQ$<br>$\frac{dR}{dt} = k_1QS - k_2R$ | $P = \frac{k_1k_3S}{k_2k_5}$<br>$Q = \frac{k_2k_4}{k_3k_1S}$<br>$R = \frac{k_4}{k_3}$ | Briat et al. (2016)<br>“N-type antithetic” |
| 14<br>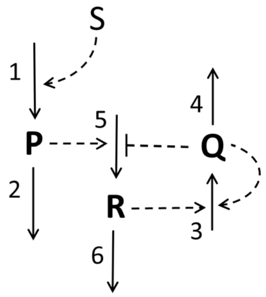 | $\frac{dP}{dt} = k_1S - k_2P$<br>$\frac{dQ}{dt} = k_3QR - k_4Q$<br>$\frac{dR}{dt} = \frac{k_5P}{1+Q} - k_6R$ | $P = \frac{k_1S}{k_2}$<br>$1 + Q = \frac{k_1k_3k_5S}{k_2k_4k_6} > 1$<br>$R = \frac{k_4}{k_3}$ | Shi et al. (2017)<br>“negative feedback loop with exponential buffer” |
| 15<br>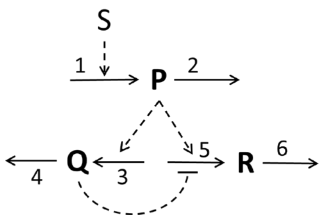 | $\frac{dP}{dt} = k_1S - k_2P$<br>$\frac{dQ}{dt} = k_3\left(\frac{P}{K_3}\right)^{m_3} - k_4Q$<br>$\frac{dR}{dt} = k_5\left(\frac{P}{K_5}\right)^{m_5}\left(\frac{L_5}{Q}\right)^{n_5} - k_6R$ | $P = \frac{k_1S}{k_2} \ll K_3, K_5$<br>$Q = \frac{k_3}{k_4}\left(\frac{k_1S}{k_2K_3}\right)^{m_3} \gg L_5$<br>$R =$<br>$\frac{k_5}{k_6}\left(\frac{k_4L_5}{k_3}\right)^{n_5}\frac{K_3^{m_3n_5}}{K_5^{m_5}}\left(\frac{k_1S}{k_2}\right)^{m_5-m_3n_5}$<br>RPA if $m_3n_5 = m_5$ | Shi et al. (2017)<br>“incoherent feedforward loop” |

**Supplementary Table S1.** The *pure* signaling motifs.[a]

| Sign Pattern | Motif | Eight distinct cases | Name |
|---|---|---|---|
| 0 0 ± 0 ± ± | 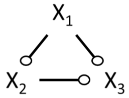  | (+ + −), (+ − +), (− + +), (− − −)<br>(− − +), (− + −), (+ − −), (+ + +) | IFFL<br>CFFL |
| ± 0 ± 0 ± 0 | 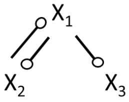  | (+ − +), (+ − −), (− + +), (− + −)<br>(+ + +), (+ + −), (− − +), (− − −) | NFLB/upper<br>PFLB/upper |
| 0 0 0 ± ± ± | 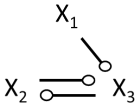  | (+ + −), (+ − −), (− + +), (− − +)<br>(+ + +), (+ − +), (− + −), (− − −) | NFLB/lower<br>PFLB/lower |
| 0 ± ± 0 0 ± | 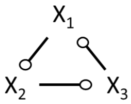  | (+ + −), (+ − +), (− + +), (− − −)<br>(+ − −), (− + −), (− − +), (+ + +) | NFL/ccw<br>PFL/ccw |
| ± 0 0 ± ± 0 | 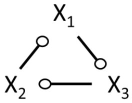  | (+ + −), (+ − +), (− + +), (− − −)<br>(+ − −), (− + −), (− − +), (+ + +) | NFL/cw<br>PFL/cw |

| Sign Pattern | Motifs | | Explanatory Notes |
|---|---|---|---|
| ± ± 0 0 ± 0<br>0 ± ± 0 ± 0 | 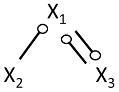  | 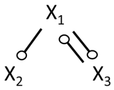  | Output node feeds back directly to input node. Regulatory node ineffective. Non-adaptive networks, according to Ma et al. (2009) |
| 0 ± 0 ± ± 0<br>0 ± 0 0 ± ± | 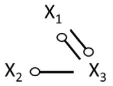  | 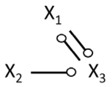  | |
| ± 0 ± 0 0 ±<br>0 0 ± ± 0 ± | 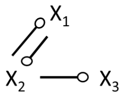  | 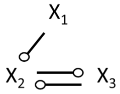  | Non-adaptive: regulatory node does not buffer the signal. |
| ± 0 0 0 ± ±<br>0 0 ± ± ± 0 | 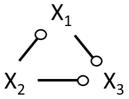  | 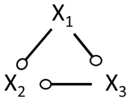  | Non-adaptive: the output node receives direct influence from input node, but no corrective action from regulatory node. |
| 0 ± ± ± 0 0<br>± ± 0 ± 0 0 | 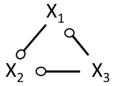  | 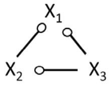  | Non-adaptive: the output node receives no influence, directly or indirectly, from input node. Therefore, no response to signal. |
| ± ± 0 0 0 ± | 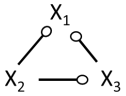  | | |
| ± ± ± 0 0 0<br>± 0 ± ± 0 0 | 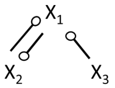  | 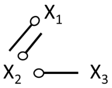  | |
| ± 0 0 ± 0 ±<br>0 ± 0 ± 0 ± | 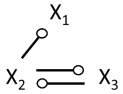  | 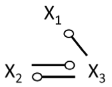  | |

[a] **Notes**: For a molecular interaction network with three links among three genes and/or proteins there are 6-choose-3 = 20 different sign patterns with three 0's and three ±'s, listed here. The sign patterns are translated into a motif diagram, where the connector ( *j* –o *i* ) indicates that species *j* either activates or inhibits species *i* ( $s_{ij} = \text{sign}(\omega_{ij}) = +$ for activation, − for inhibition). The first five sign patterns are potentially adaptive motifs, with common names: IFFL, incoherent feedforward loop; CFFL, coherent feedforward loop; NFLB, negative feedback loop with buffer node; PFLB, positive feedback loop with buffer node; NFL, three-component negative feedback loop; PFL, three-component positive feedback loop; ccw, counterclockwise; cw, clockwise. The remaining 15 sign patterns are thought to be non-adaptive for the reasons given. In addition, CFFLs and PFLBs do not adapt on their own.

**Supplementary Table S2.** NFLB-1 topologies (1X3X3X) macro-mutate predominantly into high-scoring IFFL-1 + NFLB-1 topologies (1X3X31).

| | IFFL-1 + NFLB-1 topologies | | | | | | | | | |
|---|---|---|---|---|---|---|---|---|---|---|
| NFLB-1 Topologies | 113131 | 113231 | 113331 | 123131 | 123231 | 123331 | 133131 | 133231 | 133331 | Others |
| 113132 | 0 | 0 | 0.001 | 0 | **0.954** | 0.024 | 0.001 | 0.009 | 0.004 | 0.006 |
| 113133 | 0 | 0.004 | 0.001 | 0 | **0.957** | 0.008 | 0 | 0.022 | 0.001 | 0.007 |
| 113232 | 0 | 0 | 0 | 0 | 0.006 | **0.385** | 0 | **0.329** | **0.277** | 0.004 |
| 113233 | 0 | 0.001 | 0.002 | 0.001 | **0.651** | **0.331** | 0 | 0.007 | 0 | 0.006 |
| 113332 | NA | NA | NA | NA | NA | NA | NA | NA | NA | NA |
| 113333 | 0 | 0 | 0 | 0.010 | 0.110 | 0.108 | 0.000 | 0.018 | **0.754** | 0 |
| 123132 | 0 | 0 | 0 | 0 | 0.055 | 0.010 | 0.001 | 0.015 | **0.917** | 0.001 |
| 123133 | 0.001 | 0.007 | 0 | **0.459** | **0.214** | 0.011 | 0.043 | **0.161** | 0.097 | 0.007 |
| *123232* | 0 | 0 | 0 | 0.005 | **0.189** | 0.008 | 0.001 | **0.788** | 0.006 | 0.005 |
| 123233 | 0 | 0 | 0 | 0.001 | **0.321** | 0.019 | 0.001 | **0.652** | 0.007 | 0 |
| 123332 | 0 | 0 | 0.000 | 0.001 | **0.210** | **0.352** | 0.001 | 0.004 | **0.431** | 0 |
| 123333 | 0 | 0 | 0 | 0.001 | **0.976** | 0.015 | 0 | 0.007 | 0.002 | 0 |
| 133132 | 0 | 0 | 0.002 | 0.005 | 0.020 | **0.359** | 0 | 0.106 | **0.505** | 0.002 |
| 133133 | 0.001 | 0 | 0 | **0.471** | 0.003 | 0 | **0.476** | 0.030 | 0.014 | 0.005 |
| 133232 | 0 | 0 | 0 | 0 | 0.003 | 0.004 | 0.002 | **0.543** | **0.447** | 0 |
| 133233 | NA | NA | NA | NA | NA | NA | NA | NA | NA | NA |
| 133332 | 0 | 0.000 | 0.000 | 0.001 | **0.320** | **0.280** | 0.000 | **0.393** | 0.004 | 0.001 |
| 133333 | 0 | 0 | 0 | 0.000 | **0.278** | **0.346** | 0 | **0.206** | **0.169** | 0 |

The *pure* NFLB-1 (123232) is marked in *italics*. Bold percentages indicate cases where an IFFL-1 + NFLB-1 topology occupies more than 15% of the high-scoring sample in a macro-mutation run. Percentages bordered in red indicate cases where a topology occupies more than 50% of the high-scoring sample. NA means no high-scoring topologies were found.

**Supplementary Table S3.** NFLB-3 topologies (XXX331) macro-mutate predominantly into high-scoring IFFL-1 + NFLB-1 topologies (1X3X31).

| | IFFL-1 + NFLB-1 Topologies | | | | | | | | | |
|---|---|---|---|---|---|---|---|---|---|---|
| **NFLB-3 Topologies** | 113131 | 113231 | 113331 | 123131 | 123231 | 123331 | 133131 | 133231 | 133331 | Others |
| 111331 | 0.001 | 0.004 | **0.564** | 0 | **0.372** | 0.047 | 0 | 0.007 | 0 | 0.005 |
| 112331 | 0 | 0 | 0.009 | 0 | | | 0.001 | 0.007 | 0.005 | 0.006 |
| 121331 | 0 | 0.001 | 0 | 0.005 | | | 0 | 0.006 | 0 | 0.007 |
| 122331 | 0 | 0 | 0.002 | 0 | 0.038 | | 0 | 0 | 0.006 | 0.006 |
| 131331 | 0 | 0 | 0 | 0.001 | 0.031 | | 0 | 0 | 0.006 | 0.005 |
| 132331 | 0 | 0.001 | 0.002 | 0.001 | 0.031 | | 0.001 | 0.001 | **0.247** | 0.025 |
| 211331 | 0 | 0 | 0 | 0 | 0 | 0.003 | 0 | **0.409** | **0.588** | 0 |
| 212331 | 0 | 0.003 | 0 | 0.001 | 0.001 | 0.002 | 0.001 | 0 | 0 | **0.992**[a] |
| 221331 | 0 | 0.003 | 0 | 0.001 | 0.109 | | 0 | 0.001 | 0.005 | 0.005 |
| *222331* | 0 | 0 | 0 | 0.002 | 0.004 | | 0 | 0.001 | 0.005 | 0.001 |
| 231331 | 0 | 0.002 | 0 | 0 | | 0.029 | 0 | 0.006 | 0.002 | 0.002 |
| 232331 | 0 | 0.003 | 0.001 | 0 | 0.012 | 0.008 | 0 | 0.021 | | 0.003 |
| 312331 | 0 | 0 | 0 | 0 | 0.006 | 0.006 | 0.002 | | | 0.002 |
| 322331 | 0 | 0.001 | 0.003 | 0.007 | 0.028 | | 0 | 0.042 | 0.005 | 0.002 |
| 332331 | 0.001 | 0.004 | 0 | 0 | | | 0 | **0.429** | 0.003 | 0.013 |

The *pure* NFLB-3 (222331) is marked in *italics*. Bold percentages indicate cases where an IFFL-1 + NFLB-1 topology occupies more than 15% of the high-scoring sample in a macro-mutation run. Percentages bordered in red indicate cases where a topology occupies more than 50% of the high-scoring sample. NA means no high-scoring topologies were found.

[a]In this case, the predominant endpoints are IFFL-1 only (213231, 223231, 323231) and IFFL-1 + NFLB-3 (323331 and 333331).

**Supplementary Table S4.** NFLB-2 topologies (3X1X3X) macro-mutate predominantly to high-scoring IFFL-1 + NFLB-1 topologies (1X3X31). *Italic*: the *pure* NFLB-2 (321232); **bold**: more than 15% of cases in an IFFL-1 + NFLB-1 topology. NA means no high-scoring topologies were found.

| NFLB-2 | IFFL-1 + NFLB-1 Topologies | | | | | | | | | |
|---|---|---|---|---|---|---|---|---|---|---|
| Topologies | 113131 | 113231 | 113331 | 123131 | 123231 | 123331 | 133131 | 133231 | 133331 | Others |
| 311131 | 0 | 0.005 | **0.317** | | | 0.045 | 0 | 0.003 | 0 | 0.011 |
| 311132 | 0.001 | 0 | 0 | | 0.015 | 0.002 | 0.117 | 0.005 | 0.004 | 0.016 |
| 311231 | 0.001 | 0 | 0 | 0.128 | 0.015 | 0.002 | 0 | 0.002 | 0 | **0.853**[a] |
| 311232 | 0 | 0 | 0 | 0 | 0.017 | **0.326** | 0 | 0.010 | **0.646** | 0.002 |
| 311331 | 0 | 0.001 | 0 | 0 | 0.006 | 0 | 0.003 | **0.946** | 0.011 | 0.033 |
| 311332 | NA | NA | NA | NA | NA | NA | NA | NA | NA | NA |
| 321131 | 0.001 | 0.006 | 0.004 | 0 | **0.399** | **0.578** | 0 | 0.001 | 0.004 | 0.008 |
| 321132 | NA | NA | NA | NA | NA | NA | NA | NA | NA | NA |
| 321231 | NA | NA | NA | NA | NA | NA | NA | NA | NA | NA |
| *321232* | 0 | 0 | 0 | 0 | 0 | 0 | 0 | 0 | 0 | **1.000**[b] |
| 321331 | 0 | 0.001 | 0.002 | 0.013 | **0.293** | **0.682** | 0 | 0.001 | 0.001 | 0.007 |
| 321332 | 0.005 | 0.003 | 0 | 0.016 | **0.635** | **0.328** | 0 | 0.003 | 0.003 | 0.006 |
| 331131 | **0.527** | 0.003 | 0.005 | **0.448** | 0.005 | 0.002 | 0.003 | 0.002 | 0 | 0.005 |
| 331132 | 0 | 0.004 | **0.501** | 0.001 | 0.116 | **0.331** | 0 | 0 | 0.037 | 0.009 |
| 331231 | 0.002 | **0.220** | 0.002 | 0 | 0.005 | 0.008 | 0.001 | **0.230** | **0.520** | 0.011 |
| 331232 | NA | NA | NA | NA | NA | NA | NA | NA | NA | NA |
| 331331 | NA | NA | NA | NA | NA | NA | NA | NA | NA | NA |
| 331332 | 0 | 0.001 | 0 | 0 | **0.973** | 0.004 | 0 | 0.018 | 0 | 0.005 |

[a]The dominant topologies in this case are *uncoupled* NFLB-1: 133232, 123232 and 123132.

[b]The dominant topologies in this case are IFFL-4 + NFLB-2: 331333, 321233 and 321133.

**Supplementary Table S5.** NFLB-4 topologies (XXX133) macro-mutate predominantly to high-scoring IFFL-1 + NFLB-1 topologies (1X3X31). *Italic*: the *pure* NFLB-2 (321232); **bold**: more than 15% of cases in an IFFL-1 + NFLB-1 topology. NA means no high-scoring topologies were found.

| NFLB-4 Topologies | IFFL-1 + NFLB-1 Topologies | | | | | | | | | |
|---|---|---|---|---|---|---|---|---|---|---|
| | 113131 | 113231 | 113331 | 123131 | 123231 | 123331 | 133131 | 133231 | 133331 | Others |
| 112133 | 0 | 0 | 0 | 0 | 0 | 0.003 | 0 | 0.008 | **0.989** | 0 |
| 122133 | 0 | 0 | 0 | 0 | 0.015 | 0.001 | 0 | **0.974** | 0.009 | 0.001 |
| 132133 | 0 | 0 | 0.001 | 0.004 | **0.878** | 0.006 | 0 | 0.007 | 0.004 | 0.101 |
| 212133 | 0 | 0 | 0 | 0.003 | 0.096 | 0.004 | 0 | 0.001 | 0 | **0.896**[a] |
| 213133 | 0.005 | 0 | 0 | **0.644** | **0.329** | 0.005 | 0.011 | 0.003 | 0.002 | 0.003 |
| 223133 | NA | NA | NA | NA | NA | NA | NA | NA | NA | NA |
| *222133* | 0 | 0 | 0 | 0 | 0 | 0 | 0 | 0 | 0 | **1.000**[b] |
| 232133 | 0 | 0.003 | 0.051 | 0 | **0.436** | **0.503** | 0 | 0.001 | 0.006 | 0 |
| 233133 | 0 | 0 | 0 | 0.063 | 0 | 0.003 | **0.905** | 0.018 | 0.003 | 0.010 |
| 312133 | 0 | 0 | 0 | 0 | 0.006 | 0.014 | 0 | **0.250** | **0.729** | 0.001 |
| 313133 | 0 | 0 | 0 | 0 | 0.007 | 0.003 | **0.254** | **0.707** | 0.026 | 0.002 |
| 322133 | 0.001 | 0.004 | 0.001 | 0 | **0.476** | 0.001 | 0.001 | **0.198** | **0.313** | 0.005 |
| 323133 | 0 | 0 | 0 | 0 | 0 | 0.013 | 0 | 0.008 | **0.975** | 0.004 |
| 332133 | 0 | 0 | 0 | 0.001 | 0.013 | 0.119 | 0 | **0.533** | **0.331** | 0.003 |
| 333133 | 0 | 0 | 0.001 | 0.002 | **0.606** | **0.377** | 0 | 0.005 | 0.007 | 0.002 |

[a]The dominant topologies in this case are *uncoupled* NFLB-1: 123332 and 123232.

[b]The dominant topologies in this case are IFFL-4 + NFLB-2: 331133 and 321133.

**Supplementary Table S6.** The percentage change in average score <*Z*> going from an *uncoupled* NFLB-1 topology to a coupled NFLB-1 + IFFL-1 topology. Note: the last digit changes to 1 in each case. The average change overall is +52%.

| **NFLB-1 only** | | **NFLB-1 + IFFL-1** | | **Percentage** |
|---|---|---|---|---|
| **Code** | **< *Z* >** | **Code** | **< *Z* >** | **Change** |
| 113132 | 9.35 | 113131 | 16.87 | 80 |
| 113133 | 8.63 | 113131 | 16.87 | 95 |
| 113232 | 10.32 | 113231 | 16.26 | 58 |
| 113233 | 8.73 | 113231 | 16.26 | 86 |
| 113332 | 10.69 | 113331 | 16.10 | 51 |
| 113333 | 8.97 | 113331 | 16.10 | 79 |
| 123132 | 12.06 | 123131 | 16.92 | 40 |
| 123133 | 10.46 | 123131 | 16.92 | 62 |
| 123232 | 9.80 | 123231 | 17.15 | 75 |
| 123233 | 10.55 | 123231 | 17.15 | 63 |
| 123332 | 12.29 | 123331 | 16.76 | 36 |
| 123333 | 10.71 | 123331 | 16.76 | 56 |
| 133132 | 15.40 | 133131 | 17.68 | 15 |
| 133133 | 13.20 | 133131 | 17.68 | 34 |
| 133232 | 15.00 | 133231 | 17.03 | 14 |
| 133233 | 12.48 | 133231 | 17.03 | 36 |
| 133332 | 14.29 | 133331 | 16.77 | 17 |
| 133333 | 11.99 | 133331 | 16.77 | 40 |

**Supplementary Table S7.** The percentage change in average score <*Z*> going from an *uncoupled* NFLB-2 topology to a coupled NFLB-2 + IFFL-4 topology. Note: the last digit changes to 3 in each case. The average change overall is +240%.

| **NFLB-2 only Code** | **$<Z>$** | **NFLB-2 + IFFL-4 Code** | **$<Z>$** | **Percentage Change** |
|---|---|---|---|---|
| 311131 | 1.64 | 311133 | 13.64 | 732 |
| 311132 | 6.23 | 311133 | 13.64 | 119 |
| 311231 | 4.55 | 311233 | 10.4 | 129 |
| 311232 | 4.74 | 311233 | 10.4 | 119 |
| 311331 | 2.79 | 311333 | 11.81 | 323 |
| 311332 | 4.3 | 311333 | 11.81 | 175 |
| 321131 | 1.33 | 321133 | 12.94 | 873 |
| 321132 | 7.39 | 321133 | 12.94 | 75 |
| 321231 | 5.09 | 321233 | 12.9 | 153 |
| 321232 | 7.1 | 321233 | 12.9 | 82 |
| 321331 | 4.55 | 321333 | 12.53 | 175 |
| 321332 | 4.42 | 321333 | 12.53 | 183 |
| 331131 | 4.73 | 331133 | 12.55 | 165 |
| 331132 | 7.58 | 331133 | 12.55 | 66 |
| 331231 | 4.76 | 331233 | 14.36 | 202 |
| 331232 | 4.78 | 331233 | 14.36 | 200 |
| 331331 | 2.8 | 331333 | 14.34 | 412 |
| 331332 | 8.2 | 331333 | 14.34 | 75 |

**Supplementary Table S8.** The percentage change in average score <*Z*> going from an *uncoupled* NFLB-3 topology to a coupled NFLB-3 + IFFL-1 topology. Note: the third digit changes to 3 in each case. The average change overall is +290%.

| **NFLB-3 only** Code | <br>**< *Z* >** | **NFLB-3 + IFFL-1** Code | <br>**< *Z* >** | **Percentage Change** |
|---|---|---|---|---|
| 111331 | 2.13 | 113331 | 16.1 | 656 |
| 112331 | 5.05 | 113331 | 16.1 | 219 |
| 121331 | 4.19 | 123331 | 16.76 | 300 |
| 122331 | 5.67 | 123331 | 16.76 | 196 |
| 131331 | 3.91 | 133331 | 16.77 | 329 |
| 132331 | 5.67 | 133331 | 16.77 | 196 |
| 211331 | 2.4 | 213331 | 10.93 | 355 |
| 212331 | 3.39 | 213331 | 10.93 | 222 |
| 221331 | 2.49 | 223331 | 9.34 | 275 |
| 222331 | 3.13 | 223331 | 9.34 | 198 |
| 231331 | 2.77 | 233331 | 14.99 | 441 |
| 232331 | 2.96 | 233331 | 14.99 | 406 |
| 311331 | 2.8 | 313331 | 10.33 | 269 |
| 312331 | 3.35 | 313331 | 10.33 | 208 |
| 321331 | 4.55 | 323331 | 14.91 | 228 |
| 322331 | 3.36 | 323331 | 14.91 | 344 |
| 331331 | 2.8 | 333331 | 9.19 | 228 |
| 332331 | 3.11 | 333331 | 9.19 | 195 |

**Supplementary Table S9.** The percentage change in average score <*Z*> going from an *uncoupled* NFLB-4 topology to a coupled NFLB-4 + IFFL-4 topology. Note: the third digit changes to 1 in each case. The average change overall is +330%.

| **NFLB-4 only** | | **NFLB-4 + IFFL-4** | | **Percentage** |
|---|---|---|---|---|
| **Code** | **< *Z* >** | **Code** | **< *Z* >** | **Change** |
| 112133 | 2.74 | 111133 | 12.48 | 355 |
| 113133 | 8.63 | 111133 | 12.48 | 45 |
| 122133 | 2.68 | 121133 | 11.96 | 346 |
| 123133 | 10.46 | 121133 | 11.96 | 14 |
| 132133 | 2.59 | 131133 | 12.57 | 385 |
| 133133 | 13.2 | 131133 | 12.57 | −5 |
| 212133 | 2.64 | 211133 | 11.41 | 332 |
| 213133 | 2.24 | 211133 | 11.41 | 409 |
| 222133 | 2.64 | 221133 | 13.67 | 418 |
| 223133 | 2.27 | 221133 | 13.67 | 502 |
| 232133 | 2.12 | 231133 | 11.27 | 432 |
| 233133 | 2.43 | 231133 | 11.27 | 364 |
| 312133 | 2.9 | 311133 | 13.64 | 370 |
| 313133 | 2.6 | 311133 | 13.64 | 425 |
| 322133 | 3.25 | 321133 | 12.94 | 298 |
| 323133 | 2.63 | 321133 | 12.94 | 392 |
| 332133 | 3.22 | 331133 | 12.55 | 290 |
| 333133 | 1.81 | 331133 | 12.55 | 593 |

**Supplementary Table S10.** The percentage changes in average score < $Z$ > going from an *uncoupled* IFFL-1 topology to a coupled IFFL-1 + NFLB-1 topology. Note: the first digit changes to 1 in each case. The average change overall is +23%.

| **IFFL-1 only** | | **NFLB-1 + IFFL-1** | | **Percentage** |
|---|---|---|---|---|
| **Code** | **< $Z$ >** | **Code** | **< $Z$ >** | **Change** |
| 213131 | 11.43 | 113131 | 16.87 | 48 |
| 313131 | 13.14 | 113131 | 16.87 | 28 |
| 213231 | 13.19 | 113231 | 16.26 | 23 |
| 313231 | 14.73 | 113231 | 16.26 | 10 |
| 223131 | 15.18 | 123131 | 16.92 | 11 |
| 323131 | 10.80 | 123131 | 16.92 | 57 |
| 223231 | 15.25 | 123231 | 17.15 | 12 |
| 323231 | 15.62 | 123231 | 17.15 | 10 |
| 233131 | 13.88 | 133131 | 17.68 | 27 |
| 333131 | 15.49 | 133131 | 17.68 | 14 |
| 233231 | 14.38 | 133231 | 17.03 | 18 |
| 333231 | 14.40 | 133231 | 17.03 | 18 |

**Supplementary Table S11.** The percentage changes in average score < *Z* > going from an *uncoupled* IFFL-1 topology to a coupled IFFL-1 + NFLB-3 topology. Note: the fourth digit changes to 3 in each case. The average change overall is −15%.

| IFFL-1 only Code | < *Z* > | NFLB-3 + IFFL-1 Code | < *Z* > | Percentage Change |
|---|---|---|---|---|
| 213131 | 11.43 | 213331 | 10.93 | −4 |
| 213231 | 13.19 | 213331 | 10.93 | −17 |
| 223131 | 15.18 | 223331 | 9.34 | −38 |
| 223231 | 15.25 | 223331 | 9.34 | −39 |
| 233131 | 13.88 | 233331 | 14.99 | 8 |
| 233231 | 14.38 | 233331 | 14.99 | 4 |
| 313131 | 13.14 | 313331 | 10.33 | −21 |
| 313231 | 14.73 | 313331 | 10.33 | −30 |
| 323131 | 10.8 | 323331 | 14.91 | 38 |
| 323231 | 15.62 | 323331 | 14.91 | −5 |
| 333131 | 15.49 | 333331 | 9.19 | −41 |
| 333231 | 14.4 | 333331 | 9.19 | −36 |

**Supplementary Table S12.** The percentage changes in average score < $Z$ > going from an *uncoupled* IFFL-1 topology to a coupled IFFL-1 + NFLB-1 + NFLB-3 topology. Note: first digit = 1, fourth digit = 3 in each case. The average change overall is +20%.

| **IFFL-1 only Code** | **< $Z$ >** | **NFLB-1 + NFLB-3 + IFFL-1 Code** | **< $Z$ >** | **Percentage Change** |
|---|---|---|---|---|
| 213131 | 11.43 | 113331 | 16.1 | 41 |
| 213231 | 13.19 | 113331 | 16.1 | 22 |
| 313131 | 13.14 | 113331 | 16.1 | 23 |
| 313231 | 14.73 | 113331 | 16.1 | 9 |
| 223131 | 15.18 | 123331 | 16.76 | 10 |
| 223231 | 15.25 | 123331 | 16.76 | 10 |
| 323131 | 10.8 | 123331 | 16.76 | 55 |
| 323231 | 15.62 | 123331 | 16.76 | 7 |
| 233131 | 13.88 | 133331 | 16.77 | 21 |
| 233231 | 14.38 | 133331 | 16.77 | 17 |
| 333131 | 15.49 | 133331 | 16.77 | 8 |
| 333231 | 14.4 | 133331 | 16.77 | 16 |

**Supplementary Table S13.** The percentage changes in average score < $Z$ > adding an NFLB-3 topology to a coupled IFFL-1 + NFLB-1 topology. Note: fourth digit = 3 in each case. The average change overall is −2.6%.

| NFLB-1 + IFFL-1 Code | < $Z$ > | NFLB-1 + NFLB-3 + IFFL-1 Code | < $Z$ > | Percentage Change |
|---|---|---|---|---|
| 113131 | 16.87 | 113331 | 16.1 | −4.6 |
| 113231 | 16.26 | 113331 | 16.1 | −1.0 |
| 123131 | 16.92 | 123331 | 16.76 | −0.9 |
| 123231 | 17.15 | 123331 | 16.76 | −2.3 |
| 133131 | 17.68 | 133331 | 16.77 | −5.1 |
| 133231 | 17.03 | 133331 | 16.77 | −1.5 |

**Supplementary Table S14.** The percentage changes in average score < *Z* > adding an NFLB-1 topology to a coupled IFFL-1 + NFLB-3 topology. Note: first digit = 1 in each case. The average change overall is +48%.

| **NFLB-3 + IFFL-1 Code** | **< *Z* >** | **NFLB-1 + NFLB-3 + IFFL-1 Code** | **< *Z* >** | **Percentage Change** |
|---|---|---|---|---|
| 213331 | 10.93 | 113331 | 16.1 | 47 |
| 313331 | 10.33 | 113331 | 16.1 | 56 |
| 223331 | 9.34 | 123331 | 16.76 | 79 |
| 323331 | 14.91 | 123331 | 16.76 | 12 |
| 233331 | 14.99 | 133331 | 16.77 | 12 |
| 333331 | 9.19 | 133331 | 16.77 | 82 |

**Supplementary Table S15.** The percentage changes in average score < $Z$ > going from an *uncoupled* IFFL-4 topology to a coupled IFFL-4 + NFLB-2 topology. Note: first digit = 3 in each case. The average change overall is +3.8%.

| **IFFL-4 only** | | **NFLB-2 + IFFL-4** | | **Percentage** |
|---|---|---|---|---|
| **Code** | **< $Z$ >** | **Code** | **< $Z$ >** | **Change** |
| 111233 | 11.45 | 311233 | 12.73 | 11.2 |
| 211233 | 11.89 | 311233 | 12.73 | 7.1 |
| 111333 | 14.04 | 311333 | 11.81 | −15.9 |
| 211333 | 13.25 | 311333 | 11.81 | −10.9 |
| 121233 | 12.02 | 321233 | 12.9 | 7.3 |
| 221233 | 11.96 | 321233 | 12.9 | 7.9 |
| 121333 | 13.47 | 321333 | 12.53 | −7.0 |
| 221333 | 12.15 | 321333 | 12.53 | 3.1 |
| 131233 | 13.64 | 331233 | 14.36 | 5.3 |
| 231233 | 12.19 | 331233 | 14.36 | 17.8 |
| 131333 | 13.55 | 331333 | 14.34 | 5.8 |
| 231333 | 12.61 | 331333 | 14.34 | 13.7 |

**Supplementary Table S16.** The percentage changes in average score < $Z$ > going from an *uncoupled* IFFL-4 topology to a coupled IFFL-4 + NFLB-4 topology. Note: fourth digit = 1 in each case. The average change overall is −3.2%.

| IFFL-4 only<br>Code | <br>< $Z$ > | NFLB-4 + IFFL-4<br>Code | <br>< $Z$ > | Percentage<br>Change |
|---|---|---|---|---|
| 111233 | 11.45 | 111133 | 12.48 | 9.0 |
| 111333 | 14.04 | 111133 | 12.48 | −11.1 |
| 211233 | 11.89 | 211133 | 11.41 | −4.0 |
| 211333 | 13.25 | 211133 | 11.41 | −13.9 |
| 121233 | 12.02 | 121133 | 11.96 | −0.5 |
| 121333 | 13.47 | 121133 | 11.96 | −11.2 |
| 221233 | 11.96 | 221133 | 13.67 | 14.3 |
| 221333 | 12.15 | 221133 | 13.67 | 12.5 |
| 131233 | 13.64 | 131133 | 12.57 | −7.8 |
| 131333 | 13.55 | 131133 | 12.57 | −7.2 |
| 231233 | 12.19 | 231133 | 11.27 | −7.5 |
| 231333 | 12.61 | 231133 | 11.27 | −10.6 |

**Supplementary Table S17.** The percentage changes in average score < $Z$ > going from an *uncoupled* IFFL-4 topology to a coupled IFFL-4 + NFLB-2 + NFLB-4 topology. Note: first digit = 3, fourth digit = 1 in each case. The average change overall is +3.3%.

| **IFFL-4 only** | | **NFLB-2 + NFLB-4 + IFFL-4** | | |
|---|---|---|---|---|
| **Code** | **<** $\boldsymbol{Z}$ **>** | **Code** | **<** $\boldsymbol{Z}$ **>** | **Percentage Change** |
| 111233 | 11.45 | 311133 | 13.64 | 19.1 |
| 211233 | 11.89 | 311133 | 13.64 | 14.7 |
| 111333 | 14.04 | 311133 | 13.64 | −2.8 |
| 211333 | 13.25 | 311133 | 13.64 | 2.9 |
| 121233 | 12.02 | 321133 | 12.94 | 7.7 |
| 221233 | 11.96 | 321133 | 12.94 | 8.2 |
| 121333 | 13.47 | 321133 | 12.94 | −3.9 |
| 221333 | 12.15 | 321133 | 12.94 | 6.5 |
| 131233 | 13.64 | 331133 | 12.55 | −8.0 |
| 231233 | 12.19 | 331133 | 12.55 | 3.0 |
| 131333 | 13.55 | 331133 | 12.55 | −7.4 |
| 231333 | 12.61 | 331133 | 12.55 | −0.5 |

**Supplementary Table S18.** The percentage changes in average score < *Z* > adding an NFLB-4 topology to a coupled IFFL-4 + NFLB-2 topology. Note: fourth digit = 1 in each case. The average change overall is +0.2%.

| **NFLB-2 + IFFL-4** | | **NFLB-2 + NFLB-4 + IFFL-4** | | |
|---|---|---|---|---|
| **Code** | **< *Z* >** | **Code** | **< *Z* >** | **Percentage Change** |
| 311233 | 12.73 | 311133 | 13.64 | 7.1 |
| 311333 | 11.81 | 311133 | 13.64 | 15.5 |
| 321233 | 12.9 | 321133 | 12.94 | 0.3 |
| 321333 | 12.53 | 321133 | 12.94 | 3.3 |
| 331233 | 14.36 | 331133 | 12.55 | −12.6 |
| 331333 | 14.34 | 331133 | 12.55 | −12.5 |

**Supplementary Table S19.** The percentage changes in average score < $Z$ > adding an NFLB-2 topology to a coupled IFFL-4 + NFLB-4 topology. Note: first digit = 3 in each case. The average change overall is +7.1%.

| NFLB-4 + IFFL-4 Code | < $Z$ > | NFLB-2 + NFLB-4 + IFFL-4 Code | < $Z$ > | Percentage Change |
|---|---|---|---|---|
| 111133 | 12.48 | 311133 | 13.64 | 9.3 |
| 211133 | 11.41 | 311133 | 13.64 | 19.5 |
| 121133 | 11.96 | 321133 | 12.94 | 8.2 |
| 221133 | 13.67 | 321133 | 12.94 | −5.3 |
| 131133 | 12.57 | 331133 | 12.55 | −0.2 |
| 231133 | 11.27 | 331133 | 12.55 | 11.4 |

**Supplementary Table S20.** Mean values of the six interaction coefficients from all high-scoring samples of IFFL-1 topologies.

| IFFL-1 | Interaction Coefficients | | | | | |
|---|---|---|---|---|---|---|
| Code | $\omega_{12}$ | $\omega_{13}$ | $\omega_{21}$ | $\omega_{23}$ | $\omega_{31}$ | $\omega_{32}$ |
| 113131 | -0.98 | -0.14 | 0.89 | -0.20 | 0.97 | -0.96 |
| 113231 | -1.00 | -0.13 | 0.93 | 0.00 | 0.95 | -0.98 |
| 113331 | -0.97 | -0.13 | 0.85 | 0.26 | 0.97 | -0.88 |
| 123131 | -0.99 | 0.00 | 0.88 | -0.17 | 0.97 | -0.95 |
| 123231 | -0.99 | 0.00 | 0.89 | 0.00 | 0.97 | -0.96 |
| 123331 | -1.00 | 0.00 | 0.95 | 0.16 | 0.97 | -0.99 |
| 133131 | -0.92 | 0.52 | 0.82 | -0.16 | 0.97 | -0.63 |
| 133231 | -0.85 | 0.62 | 0.54 | 0.00 | 0.97 | -0.90 |
| 133331 | -0.84 | 0.50 | 0.85 | 0.30 | 0.97 | -0.64 |
| 213131 | 0.00 | -0.13 | 0.93 | -0.29 | 0.95 | -0.97 |
| 213231 | 0.00 | -0.16 | 0.92 | 0.00 | 0.96 | -0.98 |
| 213331 | 0.00 | -0.19 | 0.56 | 0.17 | 0.66 | -0.96 |
| 223131 | 0.00 | 0.00 | 0.90 | -0.34 | 0.96 | -0.98 |
| 223231 | 0.00 | 0.00 | 0.89 | 0.00 | 0.96 | -0.98 |
| 223331 | 0.00 | 0.00 | 0.52 | 0.79 | 0.97 | -0.95 |
| 233131 | 0.00 | 0.27 | 0.90 | -0.28 | 0.95 | -0.97 |
| 233231 | 0.00 | 0.20 | 0.92 | 0.00 | 0.97 | -0.98 |
| 233331 | 0.00 | 0.29 | 0.91 | 0.34 | 0.97 | -0.98 |
| 313131 | 0.20 | -0.15 | 0.91 | -0.33 | 0.96 | -0.97 |
| 313231 | 0.27 | -0.23 | 0.92 | 0.00 | 0.97 | -0.98 |
| 313331 | 0.15 | -0.21 | 0.58 | 0.24 | 0.68 | -0.96 |
| 323131 | 0.12 | 0.00 | 0.48 | -0.20 | 0.64 | -0.95 |
| 323231 | 0.33 | 0.00 | 0.92 | 0.00 | 0.97 | -0.98 |
| 323331 | 0.27 | 0.00 | 0.91 | 0.19 | 0.97 | -0.98 |
| 333131 | 0.18 | 0.38 | 0.90 | -0.25 | 0.96 | -0.98 |
| 333231 | 0.27 | 0.36 | 0.92 | 0.00 | 0.97 | -0.98 |
| 333331 | 0.23 | 0.30 | 0.62 | 0.37 | 0.68 | -0.96 |

Red, the $\omega_{ij}$'s for the three links of the underlying IFFL-1; blue, the $\omega_{12}$'s in the NFLB-1 cases; purple, the $\omega_{23}$'s in the NFLB-3 cases.

**Supplementary Table 21.** Mean values of the six interaction coefficients from all high-scoring samples of IFFL-4 topologies.

| IFFL-4 Code | Interaction Coefficients | | | | | |
|---|---|---|---|---|---|---|
| | $\omega_{12}$ | $\omega_{13}$ | $\omega_{21}$ | $\omega_{23}$ | $\omega_{31}$ | $\omega_{32}$ |
| 111133 | -0.30 | -0.22 | -0.87 | -0.21 | 0.95 | 0.96 |
| 111233 | -0.39 | -0.16 | -0.86 | 0.00 | 0.95 | 0.96 |
| 111333 | -0.43 | -0.14 | -0.86 | 0.25 | 0.94 | 0.96 |
| 121133 | -0.29 | 0.00 | -0.88 | -0.26 | 0.95 | 0.97 |
| 121233 | -0.27 | 0.00 | -0.88 | 0.00 | 0.96 | 0.97 |
| 121333 | -0.48 | 0.00 | -0.83 | 0.22 | 0.93 | 0.96 |
| 131133 | -0.16 | 0.30 | -0.90 | -0.24 | 0.95 | 0.97 |
| 131233 | -0.31 | 0.47 | -0.89 | 0.00 | 0.95 | 0.97 |
| 131333 | -0.50 | 0.38 | -0.89 | 0.14 | 0.94 | 0.97 |
| 211133 | 0.00 | -0.21 | -0.88 | -0.25 | 0.96 | 0.97 |
| 211233 | 0.00 | -0.16 | -0.87 | 0.00 | 0.96 | 0.96 |
| 211333 | 0.00 | -0.21 | -0.88 | 0.25 | 0.95 | 0.96 |
| 221133 | 0.00 | 0.00 | -0.90 | -0.17 | 0.96 | 0.97 |
| 221233 | 0.00 | 0.00 | -0.88 | 0.00 | 0.95 | 0.97 |
| 221333 | 0.00 | 0.00 | -0.85 | 0.21 | 0.94 | 0.96 |
| 231133 | 0.00 | 0.30 | -0.87 | -0.15 | 0.95 | 0.97 |
| 231233 | 0.00 | 0.46 | -0.82 | 0.00 | 0.94 | 0.96 |
| 231333 | 0.00 | 0.37 | -0.87 | 0.20 | 0.95 | 0.96 |
| 311133 | 0.88 | -0.13 | -0.81 | -0.18 | 0.95 | 0.66 |
| 311233 | 0.23 | -0.15 | -0.83 | 0.00 | 0.95 | 0.95 |
| 311333 | 0.15 | -0.27 | -0.84 | 0.14 | 0.95 | 0.96 |
| 321133 | 0.83 | 0.00 | -0.77 | -0.49 | 0.94 | 0.70 |
| 321233 | 0.90 | 0.00 | -0.82 | 0.00 | 0.95 | 0.67 |
| 321333 | 0.85 | 0.00 | -0.84 | 0.18 | 0.94 | 0.61 |
| 331133 | 0.61 | 0.34 | -0.82 | -0.31 | 0.95 | 0.71 |
| 331233 | 0.83 | 0.29 | -0.83 | 0.00 | 0.94 | 0.57 |
| 331333 | 0.77 | 0.45 | -0.82 | 0.15 | 0.94 | 0.59 |

Green, the $\omega_{ij}$'s for the three links of the underlying IFFL-4; blue, the $\omega_{12}$'s in the NFLB-2 cases; purple, the $\omega_{23}$'s in the NFLB-4 cases.
